# Effect of Chitosan and Aloe Vera Extract Concentrations on the Physicochemical Properties of Chitosan Biofilms

**DOI:** 10.3390/polym13081187

**Published:** 2021-04-07

**Authors:** Cristiana M. P. Yoshida, Murilo S. Pacheco, Mariana A. de Moraes, Patrícia S. Lopes, Patrícia Severino, Eliana B. Souto, Classius F. da Silva

**Affiliations:** 1 Department of Pharmaceutical Sciences, Institute of Environmental, Chemical and Pharmaceutical Sciences (ICAQF), Federal University of São Paulo (UNIFESP), Diadema, São Paulo 09913-030, Brazil; cristiana.yoshida@unifesp.br (C.M.P.Y.); patslopes@hotmail.com (P.S.L.); 2 Department of Chemical Engineering, Institute of Environmental, Chemical and Pharmaceutical Sciences (ICAQF), Federal University of São Paulo (UNIFESP), Diadema, São Paulo 09913-030, Brazil; murilopch@outlook.com (M.S.P.); mamoraes@unifesp.br (M.A.d.M.); 3 University of Tiradentes (Unit), Biotechnological Postgraduate Program, Aracaju 49010-390, Brazil; patricia_severino@itp.org.br; 4 Institute of Technology and Research (ITP), Nanomedicine and Nanotechnology Laboratory (LNMed), Aracaju 49010-390, Brazil; 5 Tiradentes Institute, 150 Mt Vernon St, Dorchester, MA 02125, USA; 6 CEB-Centre of Biological Engineering, Campus de Gualtar, University of Minho, 4710-057 Braga, Portugal; 7 Department of Pharmaceutical Technology, Faculty of Pharmacy, University of Coimbra, Pólo das Ciências da Saúde, Azinhaga de Santa Comba, 3000-548 Coimbra, Portugal

**Keywords:** aloe vera, chitosan membrane, wound healing, color parameters, moisture vapor transmission rate, tensile strength

## Abstract

Chitosan films have been extensively studied as dressings in formulations for the treatment of chronic wounds. The incorporation of aloe vera (*Aloe barbadensis* Miller) into chitosan dressings could potentialize the healing process since aloe vera shows several pharmacological activities. This work aimed to evaluate the effect of aloe vera and chitosan concentrations on the physicochemical properties of the developed films. The films were obtained by casting technique and characterized with respect to their color parameters, morphology, barrier and mechanical properties, and thermal analysis. Results showed that the presence of aloe vera modified the films′ color parameters, changed barrier properties, increased fluid handling capacity (FHC), and decreased water-vapor permeability (WVP). The reduced elongation at break resulted in more rigid films. Aloe vera concentration did not significantly change film properties, but the presence of this gel increased the films’ stability at temperatures below 200 °C, showing similar behavior as chitosan films above 400 °C. The results suggest a crosslinking/complexation between chitosan and aloe vera, which combine appropriate physicochemical properties for application as wound dressing materials.

## 1. Introduction

Wound healing stands for a biological response occurring during the regeneration of damaged connective and epithelial tissues. Skin structure and functions can indeed be compromised by traumatic events, such as thermal burns, cuts, lacerations, surgical incisions, and chronic wounds (e.g., pressure ulcers or diabetic foot ulcers). Membranes designed for wound healing should have a high surface and small-sized pores that allow water and gas permeation, maintain adequate moisture of the wound and promote cell respiration. Furthermore, the pores′ size should prevent the entrance of microorganisms to the wound [1]. The main features that wound dressing membranes should depict are a dense top layer, capacity to protect the wound from physical damage and infections, and have a porous and hydrophilic inner layer that can absorb the wound exudate and provide cell adhesion and proliferation [2].

Biopolymeric films have been studied for wound dressing applications due to biocompatibility, effectiveness, and sustainable material [3,4]. Chitosan biopolymer has suitable biological and physicochemical properties for dressings applications. Chitosan is a copolymer that consists of β-(1 → 4)-linked 2-acetamido-2-deoxy-D-glucopyranose and 2-amino-2-deoxy-D-glucopyranose units. It is generally obtained by alkaline deacetylation of chitin, the main component of the exoskeleton of crustaceans, mollusks, and insects [5]. Chitosan and its derivatives have been proposed to accelerate wound healing using different formulations: Sponges, nanoparticles, scaffolds, gels, dressings, or films [6]. Besides the high film-forming capacity, chitosan stimulates cell proliferation, activates and proliferates the inflammatory cells in granular tissues, and affects macrophages′ function, thus accelerating the healing process [7,8]. Chitosan decreased the healing time and minimal scar formation during in vivo tests [9]. These properties may be strengthened by incorporating bioactive compounds like aloe vera extract, as this evergreen perennial plant exudate has been extensively used as wound healing.

We have previously developed biopolymeric films composed of copaiba oil and chitosan, by casting solvent evaporation technique, confirming the advantage of using the oleoresin to improve the mechanical, chemical, and barrier properties of developed films for application as skin dressings [10]. In the present work, we propose the exploitation of the aloe vera gel properties instead of the oleoresin. Aloe vera (*Aloe barbadensis* Miller) is an herbaceous plant that grows in tropical zones, and it does not survive under freeze temperatures. The colorless mucilaginous gel is obtained from the parenchymatous cells in the fresh leaves, and it is composed of water (99%) and mono- and polysaccharides (25% of the dry weight of the gel) [11]. Aloe vera shows many pharmacological activities such as anti-inflammatory, laxative, anti-arthritis, antibacterial, antifungal, protection against radiation, hypoglycemic effects, immunostimulant that activates macrophages and accelerates wound healing [12,13]. It contains alkaloids, amino acids, several carbohydrates and enzymes, lipids, flavonoids, terpenes, tannins and phlorotannins, steroids, anthraquinones, and saponins [14].

Langmead et al. [15] demonstrated that the antioxidant effect of aloe vera gel is associated with the presence of glutathione peroxidase, seven superoxide dismutase enzymes, and phenolic compounds. Mannose-6-phosphate and glucomannans (beta-(1-4) acetylated mannan) are, respectively, the most outstanding monosaccharide and polysaccharides in aloe vera gel. Glucomannans are mainly responsible for the healing effect, stimulating the fibroblasts′ activity and proliferation, which improve collagen production and secretion by these cells [13]. Hosseinimehr et al. [16] and Singh et al. [17] associated the healing effect of aloe vera gel to the restarting of angiogenesis, increasing blood flow, stimulating fibroblast proliferation, anti-inflammation, and antimicrobial activities and moisturizing effects. Garcia-Orue et al. [1] reported a positive outcome on treating chronic wounds using nanofibrous dressing containing a high concentration of aloe vera and polylactic-co-glycolic. As aloe vera extract shows many exciting features for wound dressings application, this study aimed to develop bioactive chitosan films incorporating different aloe vera concentrations for potential application as a wound dressing.

## 2. Materials and Methods

### 2.1. Materials

Commercial chitosan (deacetylation of approximately 82% and molar mass of about 1.47 × 10^5^ g/mol) and aloe vera extract were provided, respectively, by Brazil Polymers (São Paulo, Brazil) and Jungconsult do Brasil Produtos Naturais Ltd.a (Santa Catarina, Brazil). Chitosan was used without prior purification. Acetic acid (Diadema, São Paulo, Synth, Brazil) was used as an acidic medium, and Glycerol (Diadema, São Paulo, Synth, Brazil) was used as a plasticizer.

### 2.2. Chitosan Suspension

Chitosan (2.0% or 4.0% w/w) was dissolved in an aqueous acetic acid solution. The stoichiometric amount of acetic acid was calculated stoichiometrically to protonate all the NH_2_ groups and take into account the degree of acetylation and the sample mass. Using this base amount plus an extra 50% gave the total amount used. The chitosan was solubilized for two hours before the preparation of the film to complete chitosan solubilization.

### 2.3. Preparation of Chitosan Film

The films were prepared by the casting technique. The aloe vera extract was previously solubilized in distilled water in two different concentrations: 2.0 and 4.0% w/w. Equal volumes of chitosan and aloe vera solutions were mixed (24,000 r/min for 10 min) using homogenizer Ultra Turrax T25 (IKA, Königswinter, Germany), obtaining solutions at 1.0% and 2.0% because of the dilution. Glycerol at 1.0% w/w was added as a plasticizer agent. The resulting solutions were poured into polyethylene Petri dishes (0.21 g/cm^2^) and dried in a forced-air oven (Tecnal TE-394/2-MP, Piracicaba, São Paulo, Brazil) at 40 °C 24 h. The films were named as CH1-AV1, CH1-AV2, CH2-AV1, and CH2-AV2, in which CH1 and CH2 represent chitosan at 1.0% and 2.0% films respectively, and AV1 and AV2 represent aloe vera at 1.0% and 2.0% respectively. The final films were further characterized. Films without aloe vera were also prepared; the results were previously published by our group [10].

### 2.4. Color Analysis

Color parameters were assessed with a colorimeter (Konica Minolta, model Chroma Meter CR-400/410, Ramsey, NJ, USA) with a 30 mm diameter measuring area, using CIELab color parameters [18]. Color parameters L*, a* and b* (lightness “*L*”, red-green “*a*” and yellow-blue “*b*”, respectively) were measured. The total color difference, concerning the control film (aloe vera free film), was determined as given by Equation (1).

(1) $$∆E^{*}=\sqrt{{\left(∆L^{*}\right)}^{2}+{\left(∆a^{*}\right)}^{2}+{\left(∆b^{*}\right)}^{2}}$$

where ∆L* = L*_chitosan film_ − L*_aloe-loaded chitosan film_; ∆a* = a*_chitosan film_ − a*_aloe-loaded chitosan film_; ∆b* = b*_chitosan film_ − b*_aloe-loaded chitosan film_.

### 2.5. Scanning Electron Microscopy (SEM)

SEM images were performed on an LEO 440i scanning electron microscope (LEO Electron Microscopy Ltd., Zeiss, Jena, Germany) operating at 10 kV and 100 pcA. The cryofractured cross-sections and the surfaces of gold-sputtered films were analyzed.

### 2.6. Barrier Properties

**Figure 1:**
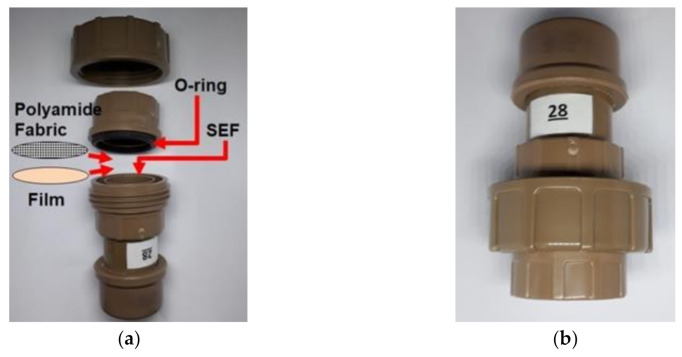
Modified Paddington cups for the determination of fluid handling capacity (FHC) (**a**); cups in inverted position (**b**).

The fluid handling capacity (FHC) of the film is the sum of the fluid absorption (ABS) by the films and moisture vapor transmission rate (MVTR). The FHC was assessed according to the BS EN 13726-1 method for dressings and hydrocolloids. Samples of each film were applied to the modified Paddington cups (Figure 1a), added 20 mL of simulated exudate fluid (SEF). SEF is an exudate-like solution that correspond to CaCl_2_ and NaCl solution containing 2.5 mmol/L Ca^+2^ and 142 mmol/L Na^+^ [19]. The cups were weighed, inverted so that the dressing came into contact with the SEF—see Figure 1b—and the set was placed in a temperature and humidity-controlled incubator to maintain an environment of 37 ± 2 °C and relative humidity (RH) below 20% for a period of 24 h

At the end of the test, the cups were removed from the incubator and were allowed to equilibrate at room temperature for 30 min before reweighing on the analytical balance. The ABS, MVTR, and FHC were calculated using Equations (2)–(4).

(2) $$\mathrm{ABS}=\frac{b-a}{time\times surface\;area}$$

(3) $$\mathrm{ABS}=\frac{b-a}{time\times surface\;area}$$

(4) FHC=ABS+MVTR

where *x* is the total system weight (cup + film + SEF solution) at the beginning of the test; *y* is the total system weight (cup + film + SEF solution) after 24 h; *a* is the film weight at the beginning of the test, and *b* is the film weight after 24 h. Five repetitions were done per experiment.

The water-vapor permeability (WVP) through films was determined gravimetrically using the method ASTM E96-95 and RH gradient of 75%. The modified Paddington cups were used with 20 g silica inside. The cups were not inverted and then placed in a desiccator containing a saturated solution of NaCl. Then, the weight gain of the cups was recorded periodically with an analytical balance. The WVP was calculated using Equation (5).

(5) $$\mathrm{WVP}=\frac{W.e}{t.A.∆P}$$

where *W* is the weight gain of the cup (g) at the time t (s), in which *W*/*t* was obtained by the slope from a plot of cup weight vs. time; *e* is the film thickness (m); *A* is the exposed area of the film (m^2^), and Δ*P* is the vapor pressure difference across the film based on the RH difference (Pa).

### 2.7. Mechanical Properties

Mechanical properties were carried out according to the ASTM D882 method. Films were cut into 10.00 cm × 2.54 cm strips. The Young’s modulus, elongation at breaking point, and tensile strength were measured using TexturePro CT V1.2 (Brookfield, CT3 50K Texturometer, Middleboro, MA, USA). The crosshead speed was set at 1 mm/s. Samples were kept in a desiccator at 75% relative humidity for 48 h before the test. At least six repetitions were assessed.

### 2.8. Thermal Analysis

Thermogravimetric analysis (TGA) and differential scanning calorimetry (DSC) were performed on chitosan films and aloe vera extract. TGA and DSC were done with a TGA-60 (Shimadzu, Kyoto, Japan) and DSC-60 (Shimadzu, Kyoto, Japan) analyzers, respectively. TGA analysis was performed with 5–10 mg samples in platinum pans in a dynamic nitrogen atmosphere (100 mL/min), in a range of 30 to 700 °C. The experiments were done at a scanning rate of 10 °C/min. For DSC analysis, samples (approx. 5–10 mg) were scanned in a sealed aluminum pan and heated to a temperature of 450 °C at 10 °C/min in a nitrogen atmosphere with a flow rate of 50 mL/min.

### 2.9. Statistical Analysis

For those analyses carried out in replicate, Tukey’s test was done to compare means, using BioEstat 5.3 (Instituto Mamirauá, Tefé, AM, Brazil).

## 3. Results and Discussion

Pure chitosan films exhibited a slightly yellow color and transparency. As described in our previous work [10], the L* parameters were 94.59 ± 0.78 and 92.07 ± 0.78 for chitosan films without aloe vera and containing, respectively, 1 and 2% of chitosan. Comparing both the L* parameter, it is clear that the aloe vera promoted a reduction in luminosity (or increase in opacity), translated by the reduction of L*. Such decrease was attributed to the increase of solids content in the films containing the extract. The aloe vera extract also promoted an increase in the films′ yellow intensity (Table 1), since the aloe vera extract solution is transparent and slightly yellow. The yellow color of the films is due to the chitosan solution color and aloe vera extract; as both concentrations increased, the yellowness (b*) increased. According to our previous work [10], the b* parameters were 8.85 ± 0.68 and 15.59 ± 1.24, respectively, for 1 and 2% of chitosan. However, when chitosan concentration was 2.0%, the b* parameters of both aloe vera concentrations were statistically the same. As depicted in Table 1, the total color difference of CH1 films ranged from 7.19 to 13.94, which is in-line with the results of Pereda et al. [20], who produced films based on tung oil/caseinate. Besides that, for CH2, the total color difference practically remained unchanged, indicating that a higher chitosan content covers the effect of color change caused by the higher concentration of aloe vera extract.

**Figure 2:**
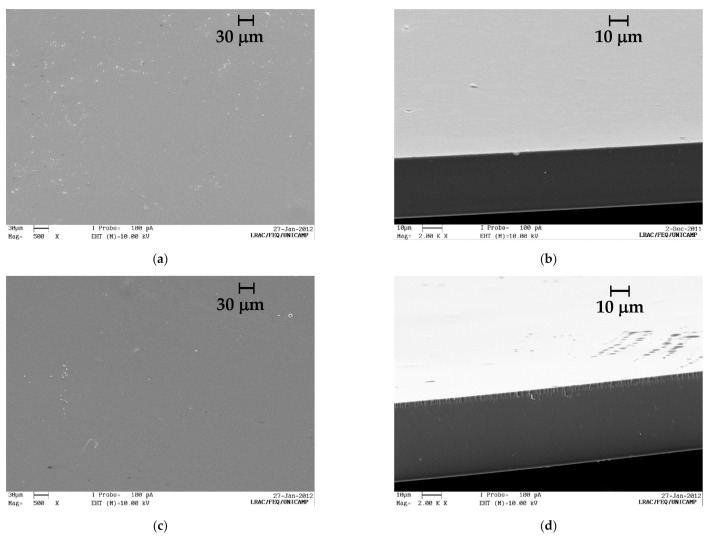
SEM images of the chitosan 2.0% films containing aloe vera extract. (**a**) Surface and (**b**) cross-section of a CH2-AV1 film at magnifications of 500× and 2000×, respectively; and (**c**) surface and (**d**) cross-section of a CH2-AV2 film at magnifications of 500× and 2000×, respectively.

Aloe vera extract was well dispersed in the chitosan suspension, and there was no macroscopic phase separation after drying. SEM micrographs allowed the description of the films′ morphology and helped us understand barrier properties. Figure 2 shows the SEM micrographs of the CH2 films containing aloe vera extract. Both the surface and cross-section presented only one homogeneous phase without any porous and cavities. The increasing of the aloe vera extract did not change the film morphology. It should be noted that the casting technique, used to produce the chitosan films, creates dense chitosan films, which means without pores. However, by hydrating the film, it is possible to create pores in the film structure when the chitosan chains are solvated. If we look at Figure 2d, the edge that delimits the surface of the film and the cross-section shows imperfections resulting from the cryofragmentation process. Our experience with chitosan films has shown structures similar to this (dense films) for pure chitosan films and chitosan films with water-soluble extracts. Aloe vera-free films showed morphology similar to the aloe vera containing films, as it can be seen in previous publications of your research group [21,22,23].

The optimal rates of wound healing can be attained by controlling the moisture content of the wound. A very dry sore may lag or hinder the healing process, but the excess of fluid can cause maceration or even infection. The best healing environment is achieved by employing an appropriate dressing; moreover, it has to be withdrawn in time to prevent maceration or adherence. Third-degree burns, unspecified granulating wounds, and donor sites yield between 3.4 and 5.1 g of exudate per 10 cm^2^ over 24 h [24].

Table 2 shows the barrier properties of aloe vera-loaded chitosan films. The results of the chitosan film without aloe vera were previously published by our group [25]. The effect of aloe vera addition was more significant for MVTR than for ABS, as the aloe vera extract increased the MVTR at about seven and ten times, respectively, for 1.0% and 2.0% of chitosan pure films. However, the aloe vera concentration did not significantly change the MVTR values of the films. Besides, when adding aloe vera extract there was a reduction in ABS values. For CH1-AV2 film, ABS capacity reduced about 20%, considering the film without the aloe. For the CH2 films, the ABS values decreased by about 30% and 60%, respectively, for AV1 and AV2 compared to the film without aloe extract. Thus, increasing aloe vera concentration, the ABS capacity of the films decreased. This suggests a possible complexing or crosslinking, since the reduction in the absorption capacity of chitosan films is common in crosslinked films, for example, with glutaraldehyde [26], with ethylene glycol diglycidyl ether [27], and with hexamethylene 1,6-bis [28].

Although the film presents a dense structure, without pores and cavities, chitosan/aloe vera films do not behave as impermeable barriers, on the contrary, they are structures that are very permeable to the simulated solution of wound exudate. This excellent permeability is due to the hydrophilicity of both chitosan and aloe vera. As FHC is one of the properties that attest the permeability of dressings. In this work, the FHC′s are even above those observed for commercial dressings described by Thomas and Young [24]. Thomas and Young [24] evaluated two film-backed foam commercial dressings (ActivHeal by Advanced Medical Solutions and Allevyn Adhesive by Smith & Nephew). The ABS values for Allevyn Adhesive and ActivHeal were 4.32 and 3.44 g/10 cm2/24 h, which were slightly higher than those reported in Table 2. Besides, the MVTR values were 12.35 and 1.67 g/10 cm^2^/24 h, respectively, for Allevyn Adhesive and ActivHeal. These commercial dressings are foam dressings; thus, such properties are usually more significant than those for film dressings, but the MVTR values were higher for most of the films listed in Table 2. Moreover, for CH2 films, the MVTR was almost three times higher.

The WVP of the aloe vera-free films were (5.38 ± 0.37) and (8.40 ± 0.47) × 10^−11^ (g/m.s.Pa), respectively for 1.0% and 2.0% chitosan films. Table 2 shows that when adding aloe vera a decrease of WVP was seen when compared to pure chitosan films, suggesting the polyelectrolyte complexation between chitosan and aloe vera. Similar results were obtained by Pereda et al. [29] for chitosan and caseinate. However, increasing aloe vera concentration at fixed chitosan concentration film did not significantly differ in the WVP values.

Chitosan film is of hydrophilic nature, in which water interacts with the chitosan matrix and increases their rate of permeation [30]. Khoshgozaran-Abras et al. [31] studied the effect of aloe vera gel on chitosan films. The authors evaluated five concentrations of aloe vera and reported the reduction of WVP by increasing the aloe vera gel ratio. The decrease was attributed to the interaction between the aloe vera compounds and the chitosan chain. Hydrophilic groups in chitosan became unavailable to water thus decreasing the WVP, which could also explain the reduction in the films′ ABS capacity containing more aloe vera extract.

Silva et al. [32] found some modifications in the FTIR spectra of chitosan/aloe-based membranes, which suggest that chitosan and aloe vera could present specific interaction. The cationic chitosan could interact with some proteins and anionic polysaccharides of the aloe vera. Many studies about chitosan films describe the polyelectrolyte complexation between chitosan and other molecules, e.g., sodium caseinate [29], alginate [33], xanthan gum [30], and soy protein [32].

Pereda et al. [34] used FTIR analysis to confirm soluble polyelectrolyte complex (PEC) formation between cationic chitosan and anionic gelatin. The authors verified that WVP of chitosan films decreased as the gelatin was added. Our results also showed a decrease in WVP by the addition of aloe vera. Chitosan films without aloe vera presented WVP values about 24% and 31% higher than those containing aloe vera, respectively, for CH1 and CH2. This behavior corroborates the potential polyelectrolyte complex (PEC) formation between chitosan and aloe vera polysaccharides.

The mechanical properties of aloe vera-loaded chitosan films are depicted in Table 3. The addition of aloe vera affected the elongation at break but did not significantly change the tensile strength of the pure chitosan films [25]. There was no statistical difference between all samples′ tensile strength, indicating that the concentration of aloe vera did not play an essential role in chitosan films′ mechanical resistance. On the other hand, the aloe vera extract decreased the elongation of the chitosan films, but the aloe vera ratio did not affect this property. This could be related to the complexation or crosslinking.

As mentioned before, a strong interaction between chitosan and aloe vera components′ chains could reduce the films′ flexibility. Besides, the increase of chitosan concentration from 1.0% to 2.0%, promoted the increase of the elongation proportionally, showing that the more chitosan present in the film, the more flexible it becomes, even in the presence of aloe vera extract. The Young modulus increased by adding aloe vera in CH1 films, but no difference was verified for CH2 films, although all the Young modulus showed values statistically in the same order. As previously reported by us [10], Young moduli for aloe vera free films were 43.9 ± 8.1 and 64.2 ± 9.3 MPa, respectively, for 1% and 2% chitosan.

Wang et al. [33] evaluated a novel chitosan-alginate polyelectrolyte complex (PEC) membrane as wound dressing material. After the coacervation between chitosan and alginate, the authors also carried out the ionic crosslinking by adding calcium chloride in three different ratios. The increase of calcium chloride slightly increased the tensile strength, the elongation at break, and the Young modulus. However, it is essential to mention that they used a very diluted chitosan solution (0.25% w/v) and alginate (0.25% w/v), thus our results exhibited different behavior. Alginate-chitosan PEC films were also studied by Pires et al. [35], in which elongation at break of the films was around 6.0%. When blended with pectin at different ratios, chitosan PEC films also presented low elongation at break values (0.5%–2.2%) [36], indicating that interactions between PEC components could decrease the flexibility of the films.

**Figure 3:**
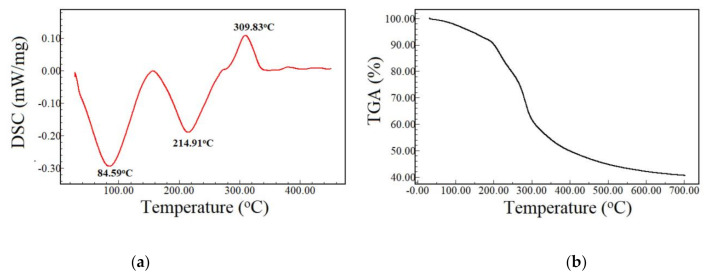
Differential scanning calorimetry (DSC) (**a**) and thermogravimetric analysis (TGA) (**b**) curves of aloe vera extract.

The effect of aloe vera gel incorporation in banana-starch/chitosan edible films was evaluated by Pinzon et al. [37]. The increase in aloe vera content resulted in a reduction in elongation at break values, attributed to a crosslinking effect caused by aloe vera components. Gallardo-Rivera et al. [38] produced a polyelectrolyte complex of chitosan, alginate, and aloe vera gel. According to the authors, aloe vera gel has a negative charge between pH 4 and 8. These results suggest that a PEC of chitosan and aloe vera by electrostatic interaction may also be drawn from our study. Figure 3 shows the DSC and TGA curves for aloe vera extract. DSC curves show an endothermic peak between 50 and 150 °C related to the vaporization of the volatile fraction. These thermic events were also assessed in TGA curves (Figure 3b). The principal mass loss of aloe vera extract occurs between 200 and 400 °C.

Garcia-Orue et al. [1] verified the presence of two endothermic peaks for the aloe vera extract at approximately 68 and 156 °C; these peaks have the temperature slightly lower than the two endothermic peaks observed in this study (84.59 °C and 214.91 °C). On the other hand, Jithendra et al. [39] found one peak for aloe vera extract at 86 °C. Although the DSC analysis was run up to 300 °C in this latter study, it was possible to observe the beginning of an exothermic peak at about 275 °C, analogous to the third peak seen in the present work.

Jithendra et al. [39] produced scaffolds of chitosan-collagen containing aloe vera. The authors found that the chitosan-collagen composite dehydration temperature remained practically unchanged compared to the pure components, chitosan, and collagen. However, this temperature increased almost 28 °C with the addition of aloe vera. This increase translates a structural reorganization of collagen by the presence of aloe vera and promoted an increase in pyrolysis resistance. The authors also verified that the loss of mass between 200 and 400 °C was significantly lower for the scaffolds with aloe vera.

**Figure 4:**
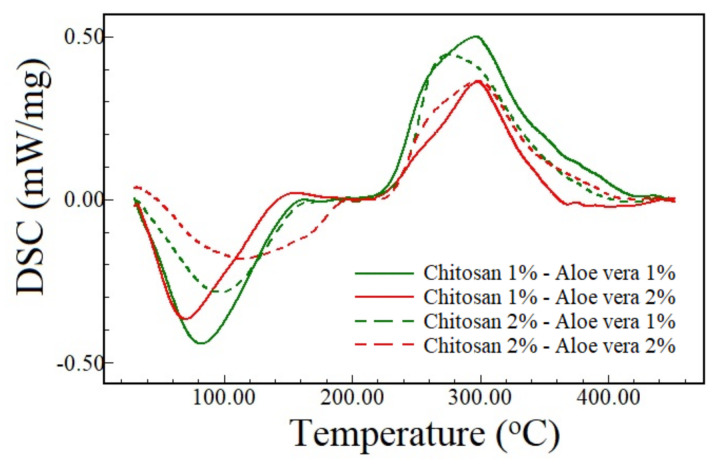
DSC run curves for 1% and 2% (w/w) chitosan films containing aloe vera extract.

The DSC of chitosan films containing aloe vera extract shows two peaks (Figure 4). The first peak, an endothermic event, corresponds to the water evaporation. These peaks were smaller and higher, respectively, for the CH1 and CH2 films. The peaks for aloe vera-free chitosan film were at 103.66 °C and 90.90 °C, respectively, for 1.0% and 2.0% chitosan [10]. According to Figure 4, these peaks were 82.17 and 70.11 °C, respectively, for CH1-AV1 and CH1-AV2. On the other hand, these peaks were at 96.78 °C and 114.54 °C, respectively, for CH2-AV1 and CH2-AV2. The second one, an exothermic event, is associated with the chitosan decomposition [40]. Ostrowska-Czubenko and Gierszewska-Druzynska [41] also verified these two peaks for chitosan films.

As the aloe vera extract increases, the chitosan decomposition temperature shifts towards higher values for CH2 films, while this effect was insignificant for CH1 films. Also, the decomposition enthalpy (area under the second peak in Figure 4) decreases. Such reductions could be attributed to the aloe vera, which is decomposed mainly about 400 °C. Thus, the interaction between chitosan and aloe vera was able to reduce the chitosan decomposition. The addition of aloe vera also promoted a substantial reduction in the enthalpy of decomposition of the films, which means that the heat released by the decomposition is lower for films containing higher aloe vera content.

**Figure 5:**
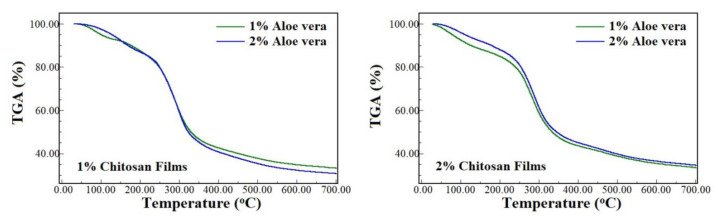
TGA curves of 1.0% and 2.0% chitosan films containing 1.0% and 2.0% aloe vera extract.

Thermogravimetric analyzes were performed to evaluate the aloe vera′s thermal stability and aloe vera/chitosan films. Together with DSC analyses, TGA analyses can help to understand and to elucidate physical or chemical interactions between compounds, in this case, between chitosan and aloe vera compounds. Thermal degradation values are presented in Table 4, and TGA curves are shown in Figure 5.

Aloe vera extract presented similar behavior for weight loss in the tested temperature range compared to chitosan powder. Besides that, up to 100 °C, aloe vera-free chitosan films′ weight loss was 12% for both chitosan concentrations. In contrast, up to 700 °C, the weight losses were 69 and 67%, respectively, for 1.0% and 2.0% chitosan concentrations, according to our previous published data [25].

The addition of aloe vera to the chitosan films promoted a reduction in the weight loss from room temperature to 200 °C but presented similar behavior in the range of 300–700 °C compared to aloe-free chitosan films. The weight losses of AV1 and AV2 films also showed identical behavior for both CH1 and CH2 films. The primary degradation of aloe vera occurs between 200 and 400 °C, which is also the main range for chitosan film decomposition. Thus, the aloe vera addition to chitosan films seems to play an essential role in increasing the films′ stability below that temperature range. Still, above that range, the films′ degradation with or without aloe vera is very similar, regardless of the aloe vera concentration. Compared to our previous study of chitosan films containing copaiba oil, our result was identical below 200 °C, where the oil stabilized the films, but opposite above 400 °C, where the oil promoted even further degradation of the films [10].

## 4. Conclusions

In this study, we developed chitosan films with the aloe vera extract for wound healing and evaluated aloe vera’s effect on the films’ properties. Aloe vera was well dispersed in the chitosan films with no phase separation. It promoted a reduction of luminosity and an increase in the films’ yellow color. The effect of aloe vera concentration in the total color difference is more pronounced at lower chitosan concentrations. The film’s barrier properties were enhanced by the aloe vera addition, being the results adequate considering commercial dressings. The films’ absorption capacity decreased with aloe vera content, suggesting a crosslinking or complexing behavior. This behavior was also indicated by elongation at the break since it decreased with aloe vera addition to the films. Thermal analysis showed that the aloe vera made films more stable below 200 °C, regardless of aloe vera concentration. The aloe vera slightly altered the films’ physicochemical properties, and the concentration of the active did not influence the final properties. Our results suggested that the films present suitable properties for wound dressings application.

## Figures and Tables

**Table 1:** Chroma parameters (L*, a*, b*), and total color difference (ΔE*) for aloe vera-loaded chitosan films.

Sample	Chroma Parameter			Color Difference (∆E*)
	L*	a*	b*	
CH1-AV1	91.02 ± 2.73 ^a^	−4.22 ± 0.32 ^a^	14.61 ± 1.58 ª	7.19
CH1-AV2	89.73 ± 3.02 ^a,b^	−4.32 ± 0.35 ^a^	21.68 ± 1.97 ^b^	13.94
CH2-AV1	87.50 ± 1.04 ^b,c^	−3.34 ± 0.23 ^b^	26.39 ± 1.66 ^c^	12.48
CH2-AV2	86.08 ± 1.16 ^c^	−2.81 ± 0.26 ^c^	27.49 ± 2.31 ^c^	13.27

Values are mean ± SD (*n* = 10). Different superscripts within a column indicate significant differences among formulations (*p* < 0.05).

**Table 2:** Barrier properties—moisture vapor transmission rate (MVTR), absorption (ABS), fluid handling capacity (FHC), and water vapor permeation (WVP)—of the chitosan films for different concentrations of aloe vera extract.

Samples	MVTR (g/10 cm^2^/24 h)	ABS (g/10 cm^2^/24 h)	FHC (g/10 cm^2^/24 h)	WVP × 10^11^ (g/m.s.Pa)
CH1-AV1	12.19 ± 0.92 ^a^	1.61 ± 0.16 ^a^	13.80 ± 0.9 ^a^	4.50 ± 0.42 ^a^
CH1-AV2	12.74 ± 1.07 ^a^	1.30 ± 0.09 ^b^	14.04 ± 1.1 ^a^	4.19 ± 0.07 ^a^
CH2-AV1	31.90 ± 2.63 ^b^	2.52 ± 0.22 ^c^	34.42 ± 2.6 ^b^	6.56 ± 0.55 ^b^
CH2-AV2	31.85 ± 0.92 ^b^	1.42 ± 0.14 ^a,b^	33.27 ± 0.9 ^b^	6.24 ± 0.27 ^b^

Different superscripts within a column indicate significant differences among formulations (*p* < 0.05).

**Table 3:** Effect of the concentrations of chitosan and aloe vera extract on Young modulus, tensile strength, and elongation at the breaking point for the emulsified films.

Samples	Young Modulus (MPa)	Tensile Strength (*n*/m^2^)	Elongation at Break (%)
CH1-AV1	63.3 ± 6.8 ^a^	180.5 ± 26.0 ª	2.9 ± 0.5 ^a^
CH1-AV2	61.6 ± 5.6 ^a^	151.7 ± 33.6 ª	2.7 ± 0.6 ^a^
CH2-AV1	60.6 ± 7.3 ^a^	176.9 ± 23.2 ª	6.7 ± 1.6 ^b^
CH2-AV2	68.6 ± 10.0 ^a^	189.1± 33.1 ª	5.6 ± 1.6 ^b^

Values are mean ± SD (*n* = 5). Different superscripts within a column indicate significant differences among formulations (*p* < 0.05).

**Table 4:** Percentage weight loss obtained by thermogravimetric analysis of chitosan powder, aloe vera extract, and chitosan films with aloe vera extract.

Concentration (% w/w)		Percentage of Cumulative Weight Loss (%)						
Chitosan	Aloe Vera	100 °C	200 °C	300 °C	400 °C	500 °C	600 °C	700 °C
1	1.0	5	12	40	57	62	65	67
	2.0	2	13	41	59	65	68	69
2	1.0	7	15	41	56	61	65	67
	2.0	4	12	38	54	59	62	64
Aloe vera extract		2	10	38	50	55	58	59

## Data Availability

Data are available from corresponding authors upon request.
